# A Web Platform (MOSAICO) to Design, Perform, and Assess Collaborative Clinical Scenarios for Medical Students: Viewpoint

**DOI:** 10.2196/23370

**Published:** 2021-01-26

**Authors:** Sergio Guinez-Molinos, Jaime Gonzalez Díaz, Carmen Gomar Sancho, Paulina Espinoza, Gustavo Constenla

**Affiliations:** 1 Laboratory of Biomedical Informatics School of Medicine Universidad de Talca Talca Chile; 2 Faculty of Medicine Universität de Barcelona Barcelona Spain; 3 Mahatma Gandhi Clinical Simulation Center School of Medicine Universidad de Talca Talca Chile

**Keywords:** collaborative clinical simulation, electronic simulation record, medical students, medical education, MOSAICO

## Abstract

**Background:**

The collaborative clinical simulation (CCS) model is a structured method for the development and assessment of clinical competencies through small groups working collaboratively in simulated environments. From 2016 onward, the CCS model has been applied successfully among undergraduate and graduate medical students from the Universidad de Talca, Chile; the Universität de Barcelona, Spain; and the Universidad de Vic-Manresa, Spain. All the templates for building the clinical cases and the assessment instruments with CCS were printed on paper. Considering the large number of CCS sessions and the number of participating students that are required throughout the medical degree curriculum, it is impossible to keep an organized record when the instruments are printed on paper. Moreover, with the COVID-19 pandemic, web platforms have become important as safe training environments for students and medical faculties; this new educational environment should include the consolidation and adaptation of didactic sessions that create and use available virtual cases and use different web platforms.

**Objective:**

The goal of this study is to describe the design and development of a web platform that was created to strengthen the CCS model.

**Methods:**

The design of the web platform aimed to support each phase of the CCS by incorporating functional requirements (ie, features that the web platform will be able to perform) and nonfunctional requirements (ie, how the web platform should behave) that are needed to run collaborative sessions. The software was developed under the Model-View-Controller architecture to separate the views from the data model and the business logic.

**Results:**

MOSAICO is a web platform used to design, perform, and assess collaborative clinical scenarios for medical students. MOSAICO has four modules: educational design, students’ collaborative design, collaborative simulation, and collaborative debriefing. The web platform has three different user profiles: academic simulation unit, teacher, and student. These users interact under different roles in collaborative simulations. MOSAICO enables a collaborative environment, which is connected via the internet, to design clinical scenarios guided by the teacher and enables the use of all data generated to be discussed in the debriefing session with the teacher as a guide. The web platform is running at the Universidad de Talca in Chile and is supporting collaborative simulation activities via the internet for two medical courses: (1) Semiology for third-year students (70 students in total) and (2) Medical Genetics for fifth-year students (30 students in total).

**Conclusions:**

MOSAICO is applicable within the CCS model and is used frequently in different simulation sessions at the Universidad de Talca, where medical students can work collaboratively via the internet. MOSAICO simplifies the application and reuse of clinical simulation scenarios, allowing its use in multiple simulation centers. Moreover, its applications in different courses (ie, a large part of the medical curriculum) support the automatic tracking of simulation activities and their assessment.

## Introduction

Medical education has progressed toward student-centered learning approaches that allow students to have a more active role in their learning and the development of competencies compared with classic teacher-centered approaches [1-3]. Clinical simulation (CS) [4] and computer-supported collaborative learning (CSCL) [5] are two of these paradigms that provide significant student-centered benefits [6,7]. ﻿ Both methodologies can improve teamwork processes (eg, communication, coordination, and cooperation), and their implementation has been associated with improvements in the quality of patient care [8].

The CSCL environment empowers students to collaborate through technology, which positively influences their learning [7,9]. With the COVID-19 pandemic, the new educational environment includes consolidation and adaptation of didactic sessions creating and using available virtual cases [10] and taking advantage of different web platforms [11]. In particular, for medical students, some software helps to build clinical cases based on data from real patients [12], applying artificial intelligence [13], or recommendations by experts [14].

The collaborative clinical simulation (CCS) model is a structured learning model for the acquisition and assessment of clinical competencies through small groups working collaboratively to design and perform in simulated environments supported by technology [15]. CCS is presently a comprehensive model because it contains essential considerations and recommendations from both paradigms (ie, CS and CSCL) [15].

An essential feature of the CCS model is its capability to support the collaborative design of CS cases, which applies to a large part of the medical curriculum. The cases are created for untrained medical students guided by a teacher. The process is based on what was learned in classroom sessions; it is an instance where students can learn collaboratively, integrating information in the construction of a clinical case while acquiring, reinforcing, and applying their knowledge and skills in a simulated clinical environment [16]. The medical students create a clinical case in small groups working separately. ﻿Each group is given 60 minutes for designing the simulated scenarios, with roles, medical records, nursing sheets, and assessments [15]. The designer groups apply the clinical cases created in the simulation session to another group (ie, performer groups) and evaluate their performance, with templates given by the teacher, during the collaborative simulation phase. The teacher monitors all the processes, supports the design, assesses the performance group, and conducts the debriefing [17].

From 2016 onward, the CCS model has been applied successfully among undergraduate and graduate medical students from the Universidad de Talca, Chile; the Universität de Barcelona, Spain, [17]; and the Universidad de Vic-Manresa, Spain. Throughout these CCS sessions, the templates for building the clinical cases and the assessment instruments were printed on paper. With printed templates, the students could design a case collaboratively in small groups, take notes, create laboratory tests, and assess their performance between peers. 

Considering the large number of CCS sessions and the number of participating students that are required throughout the medical degree curriculum, it is impossible to keep a clean record when the instruments are printed on paper. Tracking simulation activity logs, scheduling simulation sessions, creating templates for assessment instruments, sharing patient data, monitoring the progress of students, and reusing clinical cases are impossible when the work is paper based. Moreover, managing all of the above information requires specialized software in order for a CS center to maintain systematized indicators, attendances, inventories, and simulation activity logs. A platform must support the management of a center with an emphasis on the teaching and learning processes with innovative tools for the collaboration between students and teachers.

To give computer support to collaborative simulation activities with the CCS model, we developed MOSAICO, a web-based platform. This platform allows for the designing, performing, and assessing of collaborative CSs through small groups working collaboratively in simulated environments supported by technology. The web platform enables a collaborative technological environment where each group works independently, while connected by the internet, to design clinical scenarios guided by the teacher, perform in the simulated clinical scenarios, and use all data generated for discussing and closing their learning gaps in the debriefing session with the teacher as a guide. 

Considering the importance of tracking the progress of medical students adequately, the web platform was created to strengthen the CCS model [15], assist in the co-construction of shared understanding, and research the interactions between participants in simulation activities with technology [18]. The development of an electronic registry for CSs has the potential to positively affect the medical trainee workflow through different mechanisms, including reducing time spent in design, accessing cases, easing the process of data retrieval, providing greater remote access, ﻿and monitoring the progress of medical students [19].

The benefits of using simulations and tracking the progress of students in health care, construction, engineering, aviation, natural resources, and the military are widely documented [20]. The capabilities offered by simulations have created unlimited opportunities in areas such as aviation training, where the use of simulations is realistic, safe, and cost-effective and allows for tracking all the activities of the future pilots [21]. The tracking of simulated flight hours, the competencies developed, and the facilities to build and reuse different scenarios are essential characteristics of flight simulators. Medical education, like aviation, is driven by needs; use of simulations in this context allows for the tracking of activities of medical students.

This paper aims to describe the MOSAICO web platform, which was created to facilitate and expand its application and track student progress across the curriculum.  To reach this goal, this paper reports on the requirements elicitation, design, and development process for implementing MOSAICO in the Faculty of Medicine of both the Universidad de Talca, Chile, and the Universität de Barcelona, Spain.

## Methods

The requirements elicitation was the first phase of the web platform development process. This phase is a critical aspect because it lays the foundation for all the subsequent project work, and it affects the success of the development project [22]. The CCS model [15] was the primary source of the requirements elicitation, which analyzes and documents the requirements via four phases. The process was conducted by the lead researcher of the web platform development (SGM) to ensure the quality and completeness of the CCS model.

**Figure 1:**
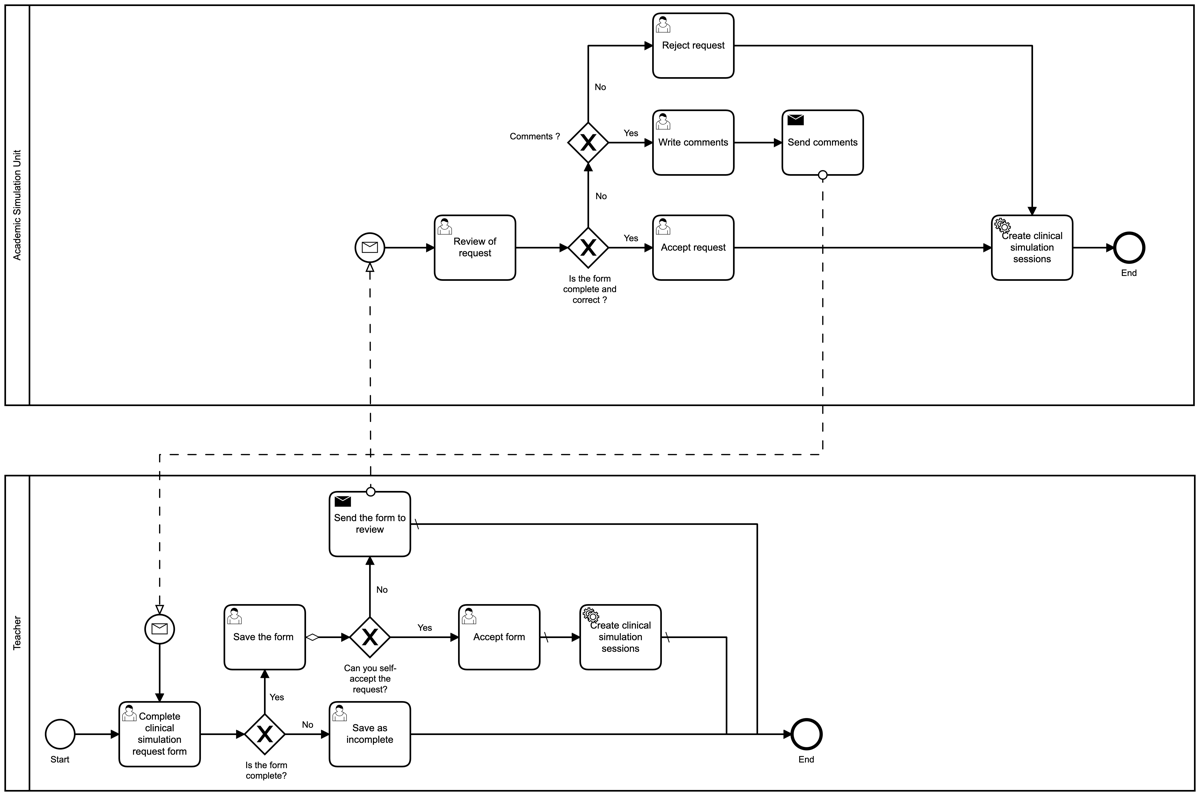
Process of scheduling a simulation session. The Business Process Model and Notation diagram of the process to request a clinical simulation that involves the collaborative work of a teacher with the academic simulation unit.

In order to complement the features and structure of the web platform, we interviewed the personnel involved in academic activities, such as medical professors, students, and support staff. The interviews were semistructured for each CCS phase, and each meeting was recorded and documented. For the requirements elicitation from academics, we used an ad hoc instrument to document and analyze all the requirements. Furthermore, we modeled each process workflow involved in the functional requirements by module (eg, schedule simulation session, create a clinical guide, and assign group) with the Business Process Model and Notation [23] standard using the free software Camunda Modeler, version 4.4 [24] (see Figure 1).

The documentation and analysis of the requirements to generate the web platform were divided into functional and nonfunctional. The functional requirements are the features that the web platform will be able to perform, such as schedule simulation, create a clinical guide, and design evaluation rubrics. Nonfunctional requirements describe how the web platform should behave, such as security, interoperability, and performance [25].

The web platform was designed to support each phase of the CCS by incorporating the functional requirements needed to run collaborative sessions. In this way, the system comprised four modules: (1) educational design, (2) students’ collaborative design, (3) collaborative simulation, and (4) collaborative debriefing. Regarding the nonfunctional requirements, usability is essential ﻿to the design of functional interfaces [26] by considering specific characteristics of the medical curriculum, technological aspects, students’ interactions, and instructional design [27,28]. Moreover, the interoperability is fundamental for sharing information between different platforms, exporting assessment instruments, sharing laboratory and multimedia tests, and reusing clinical cases.

On the other hand, the platform needs to secure users’ authentication with different roles and permissions. Security and confidentiality are essential, since small groups of students need to generate clinical cases, which are monitored by an instructor, without the other students knowing the diagnosis. For the collaboration, all the interactions between students (ie, suggestions about patient conditions, sharing laboratory and multimedia diagnostic tests, and performance assessments) can be achieved with the assistance of mobile devices (ie, tablets or smartphones). At the end of the simulation sessions (ie, collaborative debriefing), it is necessary to store the information on simulated clinical cases and the evaluations of both students and instructors as well as keeping records of collaborative debriefing sessions.

**Figure 2:**
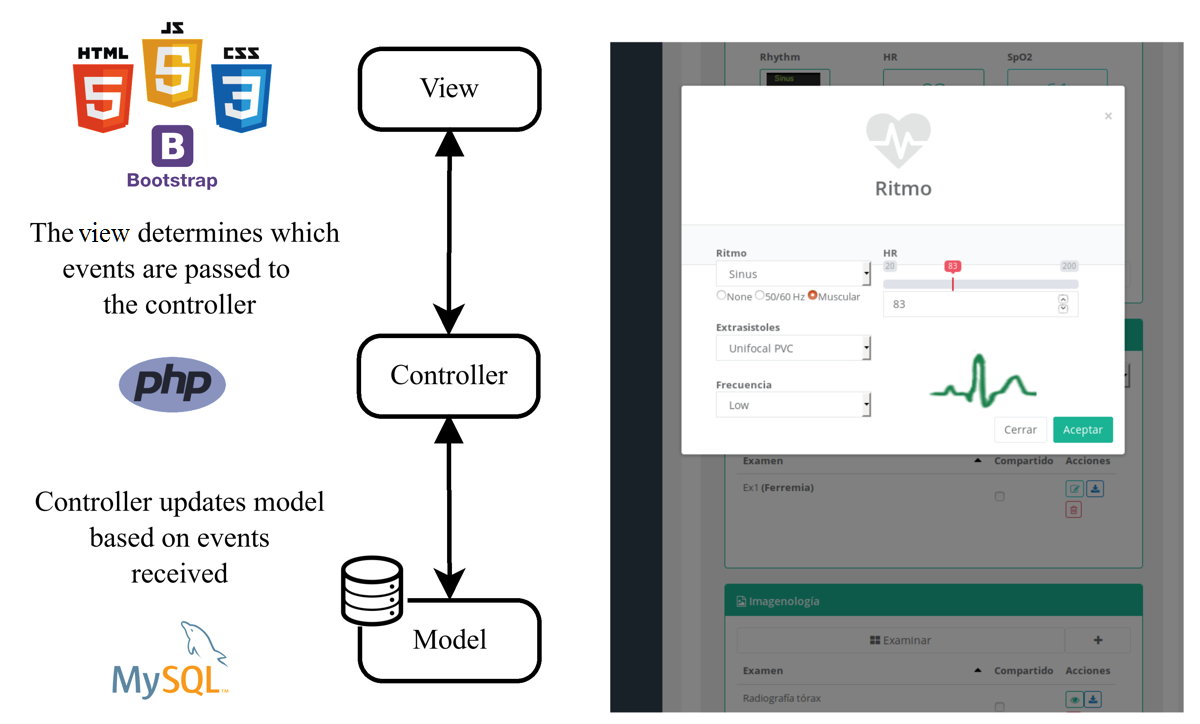
The software architecture and technologies implemented. The platform applies the Model-View-Controller architectural pattern. Controllers manage all requests from the view layer and update the model based on events or data received. The view layer renders the data sent from the model layer through a controller, and the web-based responsive interface uses technologies on a wide variety of devices, such as smartphones, tablets, or desktop computers. CSS: Cascading Style Sheets; JS: JavaScript; PHP: Hypertext Preprocessor; SQL: Structured Query Language.

After designing the web platform, we considered its development to include all the functional and nonfunctional requirements. The software was built under the Model-View-Controller architecture [29] to separate the views from the data model and the business logic. Figure 2 shows the software architecture and the technologies employed. Since usability is one of the most important nonfunctional requirements, views use web technologies, such as HTML5, JavaScript, and Cascading Style Sheets 3 (CSS 3), to ensure suitable access to different web browsers. The Bootstrap framework, version 3.7, gives the software responsive capability to fit different screen resolutions (ie, mobile and desktop devices). The model defined Hypertext Preprocessor (PHP) classes that represent the database schema and defined methods to update, select, and insert data into the database. Controllers call the model classes and use their methods to access the data. Both the model and controller were written with PHP 5.6 code. MySQL (Structured Query Language) 14.14 was used as the database management system (see Figure 2).

A full-time bioinformatics engineer (JGD) and the lead of the biomedical informatics laboratory (SGM) designed and developed the web platform. They took 12 months to create the prototype and 6 months to make modifications during the pilot application. As of 2020, the pilot application is operative for undergraduate medical students at the Universidad de Talca in Chile.

## Results

### Overview

**Figure 3:**
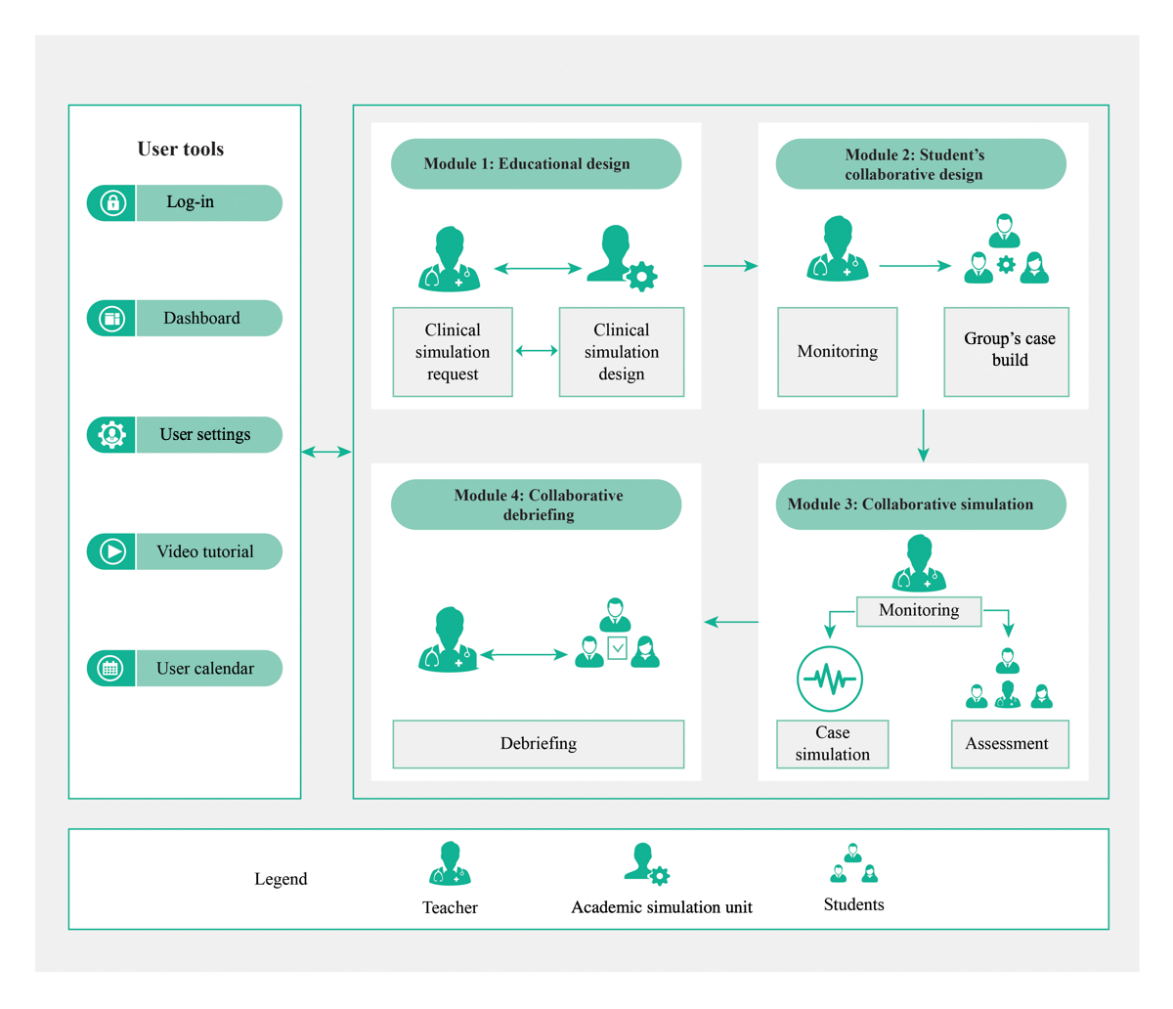
The different sequential layers designed and developed by the collaborative clinical simulation (CCS) software MOSAICO. Each module supports the CCS phases and uses technology to design, develop, and assess collaborative clinical scenarios for medical students.

MOSAICO has four modules and three different user profiles: academic simulation unit, teacher, and student. These users interact under different roles in collaborative simulations. The academic simulation unit profile includes the platform administrator, who oversees user accounts, courses, supplies, and room settings; generates reports; and works collaboratively with a teacher in order to validate and schedule simulation sessions. On the other hand, the teacher profile generates clinical guidelines with differential diagnosis and supports the platform execution with medical students. Finally, the student profile participates in modules 2, 3, and 4, which involves designing, executing, and debriefing a clinical case in small groups, collaboratively (see Figure 3). ﻿

The results of the functional requirements engineering process involved elicitation, documentation, and analysis [25], which were obtained directly from the foundational paper of the CCS model [17]. These results were complemented with different interviews and discussions about the functionalities and attributes to build a web platform for a collaborative CS. Table 1 shows the results of the most critical requirements to design the software classified by the four CCS model phases. Moreover, the nonfunctional requirements, which specify how the web platform should behave and were obtained from professors, students, and support staff, are listed in Table 1.

**Table 1:** Results of functional and nonfunctional requirements to design and develop the web platform MOSAICO for the collaborative clinical simulation (CCS) model. Functional requirements were obtained from Guinez-Molinos et al [15].

CCS model phases [15]	Platform software requirements	
	Functional	Nonfunctional
Module 1: educational design	Schedule simulation session (date and time, educational objectives, and materials) Create a clinical guide Design evaluation rubrics	Interoperability Usability Access security (authentication)
Module 2: students’ collaborative design	Record attendance Assign groups Create collaborative clinical scenarios (small groups) including: Patient history Vital signs Laboratory tests Roles Multimedia tests Monitoring student progress (online)	Interoperability Usability Access security (authorization, authentication, and privacy)
Module 3: collaborative simulation	Share patient data (laboratory and multimedia tests and vital signs) Apply evaluation rubric	Interoperability Usability Mobile usability
Module 4: collaborative debriefing	Debriefing module with: All scenarios Evaluations Videos (if possible)	Interoperability Usability Storage capacity

### Module 1: Educational Design

**Figure 4:**
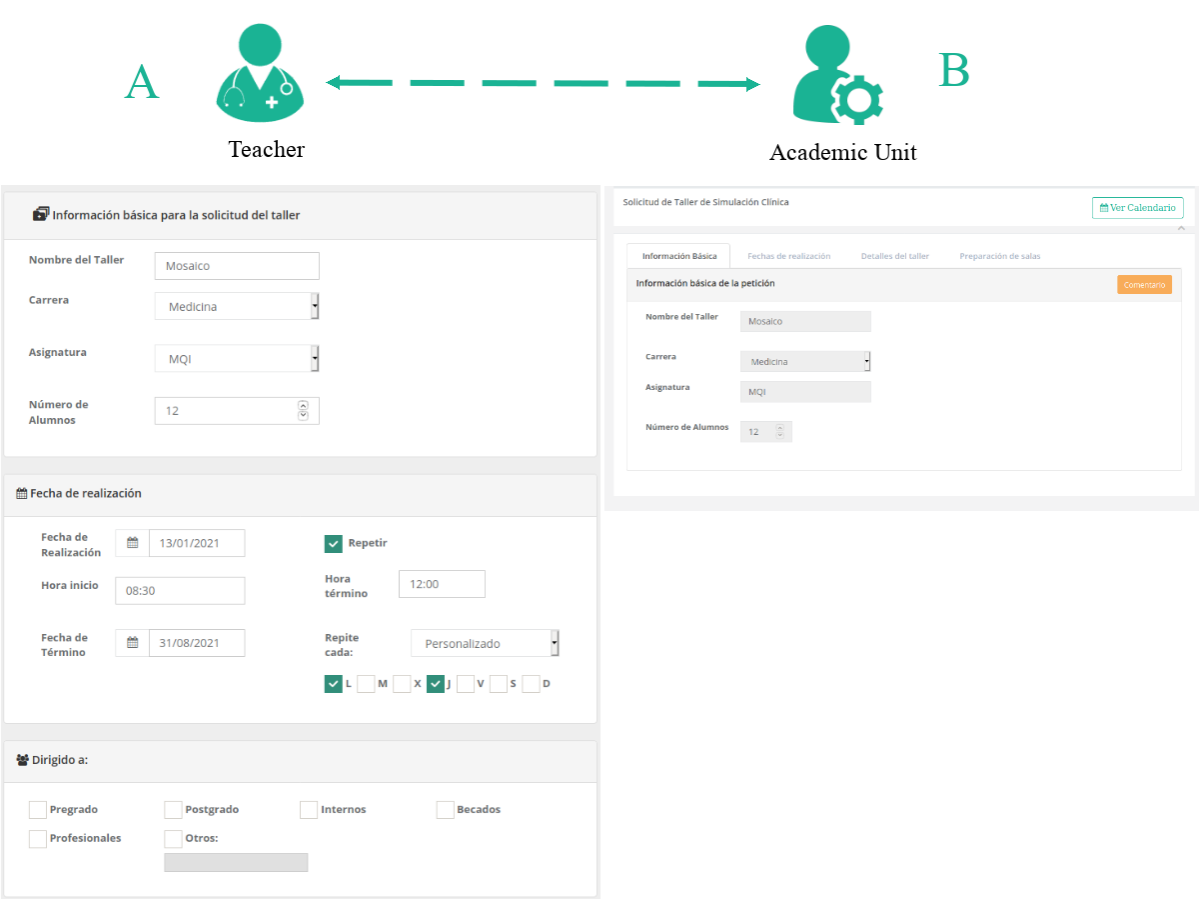
Educational design module. Form to request a clinical simulation to the academic simulation unit director (A), who reviews the request and sends comments to the teacher (B).

To schedule CS sessions, the teacher requests the academic simulation unit through the educational design module where it is possible to create, edit, review, check status, and delete the request (see Figure 4). To create a new request in MOSAICO, the teacher must define a name for the clinical session, select the audience (ie, undergraduate or graduate course, number of participants, academic degree, and competencies), propose to schedule the rooms, and create educational objectives. The teacher and the academic simulation unit profiles must design the components of the CCS (ie, objectives, materials, case scenarios, and assessment items) collaboratively ﻿according to the medical curriculum and student needs [30].

**Figure 5:**
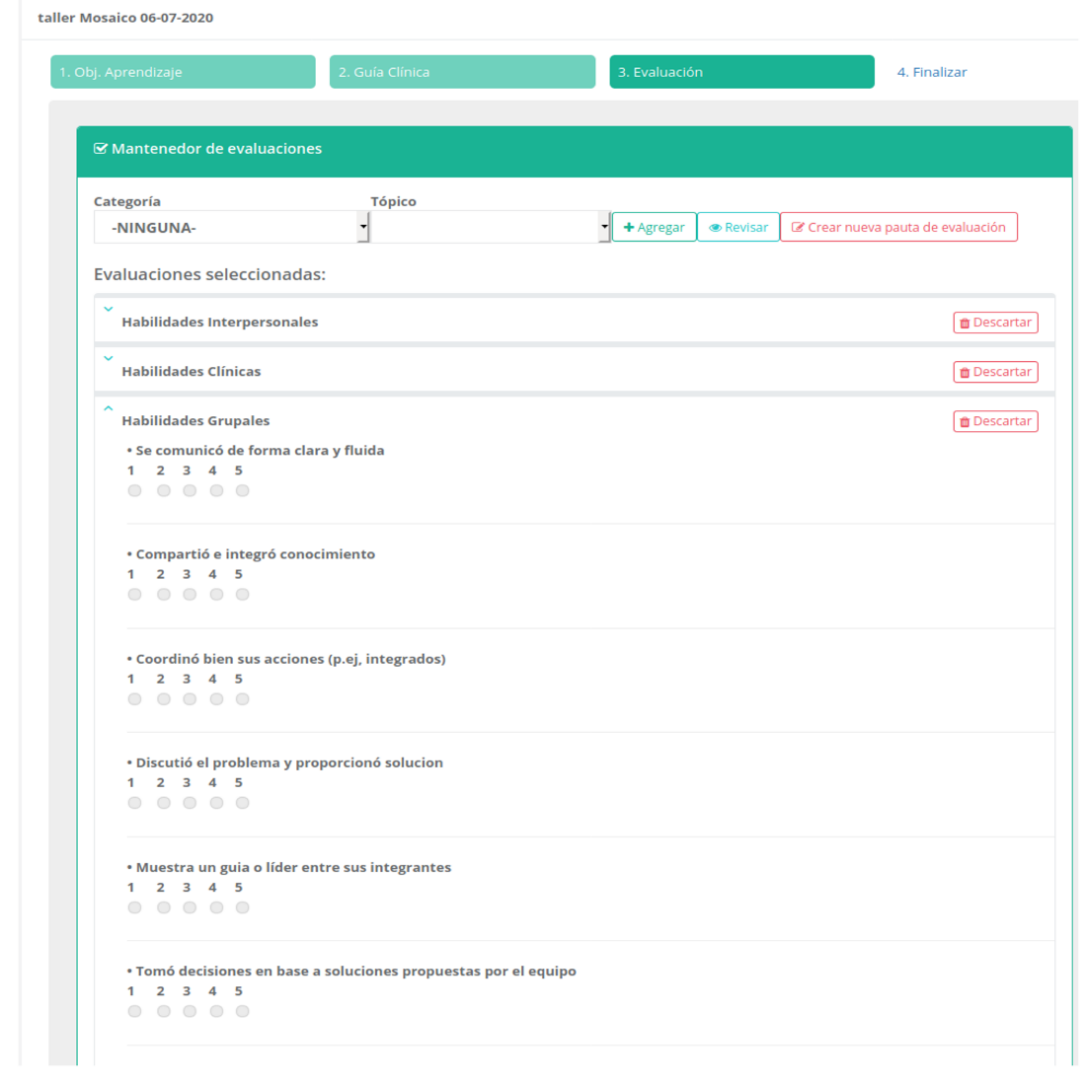
Design of the simulation session module. Once the request by the academic simulation unit is approved, the teacher can design the session with a clinical guide and rubric to assess the competencies.

In an iterative process supported by MOSAICO, the teacher and the academic simulation unit can incorporate progressive changes, which might be necessary to complete the CS request (see Figure 4) adequately. Thus, the request may take the status of incomplete (ie, in preparation), waiting (ie, sent to be reviewed by the academic simulation unit), commented (ie, reviewed by the academic simulation unit, where a teacher must make changes for it to be accepted), and accepted or rejected. Once that request has been approved and scheduled by the academic simulation unit, the teacher should upload the clinical guides, multimedia tests (eg, videos, x-rays, laboratory results, and electrocardiograms), and assessment items, based on existing templates or from scratch (see Figure 5). With this information, the system schedules a new CS session.

### Module 2: Students’ Collaborative Design

MOSAICO can track simulation activity logs of medical students, generating recorded activities [31,32]. Toward this goal, each time the medical students are about to start simulation activities, they register their attendance through a fingerprint reader; they must register their entry, select the simulation session, and register the exit when the activity has ended.

**Figure 6:**
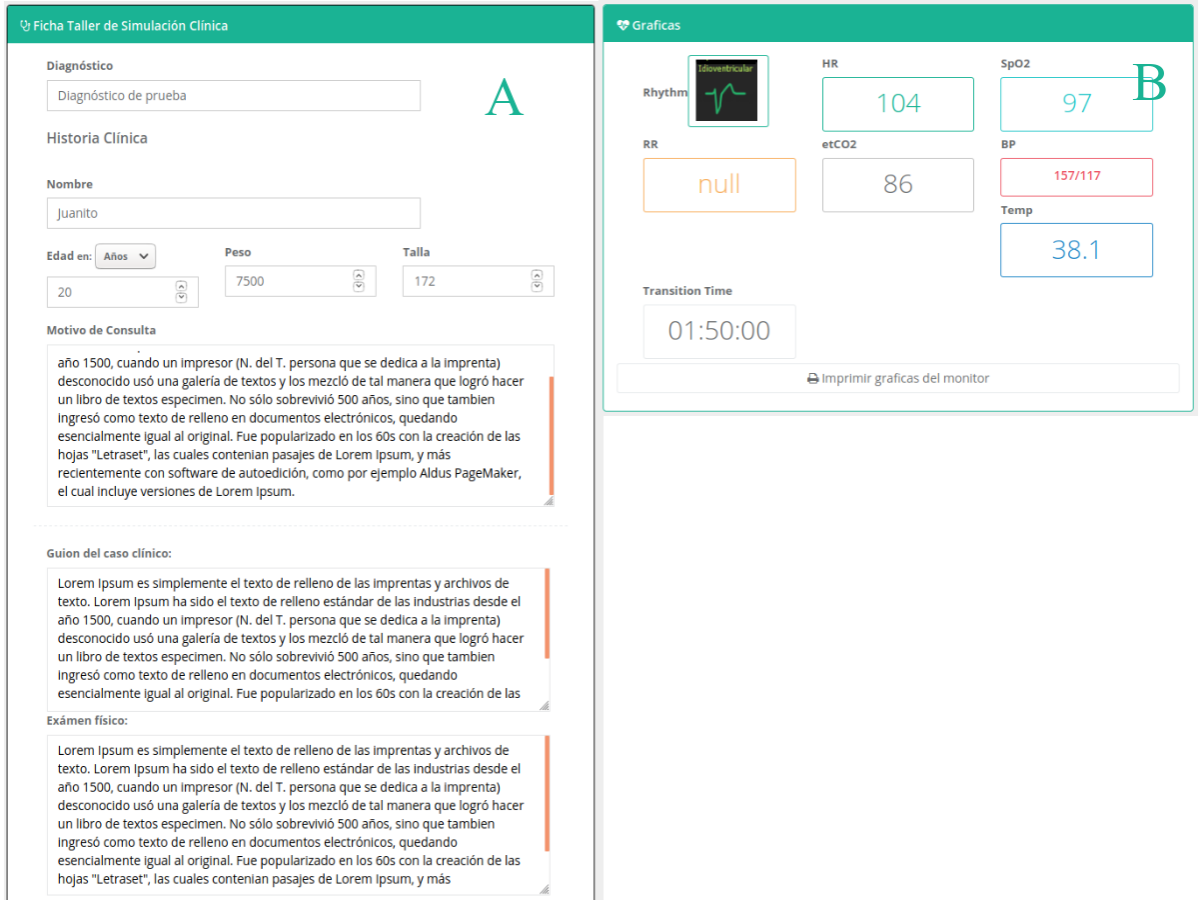
Students’ collaborative design module. (A) and (B) are the views of the web platform's graphical interface. (A) shows a view of the students' case design form, where the designer group enters information to support their case design. (B) shows a teacher's view from where he or she can monitor the students' activity remotely and in real time.

The instructor divides the students into at least three small groups of 3 to 5 students, which are deployed to different rooms [15,33]. Each group has access to a computer connected to the internet. Each group designs a clinical case in the students’ collaborative design module, according to the differential diagnosis assigned by the teacher (see Figure 6).

For the collaborative design of a clinical case, MOSAICO provides standardized templates with all the required information. The students write down relevant information about the clinical case (see Figure 6A), including age, sex, weight, size, physical exam, vital signs, laboratory tests, images, or videos (see Figure 6B). Moreover, the students describe the roles they play in the next phase (ie, collaborative simulation) for each designer group member (eg, patient, nurse, and family member).

Medical students collaboratively design a clinical case by forming small groups in person—before the COVID-19 pandemic—at the Clinical Simulation Center, where one student writes the required information with the group’s consensus on the web platform. This year, with the COVID-19 pandemic, the semiology course in medicine is using MOSAICO to teach online academic activities. This web platform allows students to design a clinical case collaboratively via the internet while at home. One of MOSAICO’s strengths is that it allows communication between students in person or online through the web platform.

**Figure 7:**
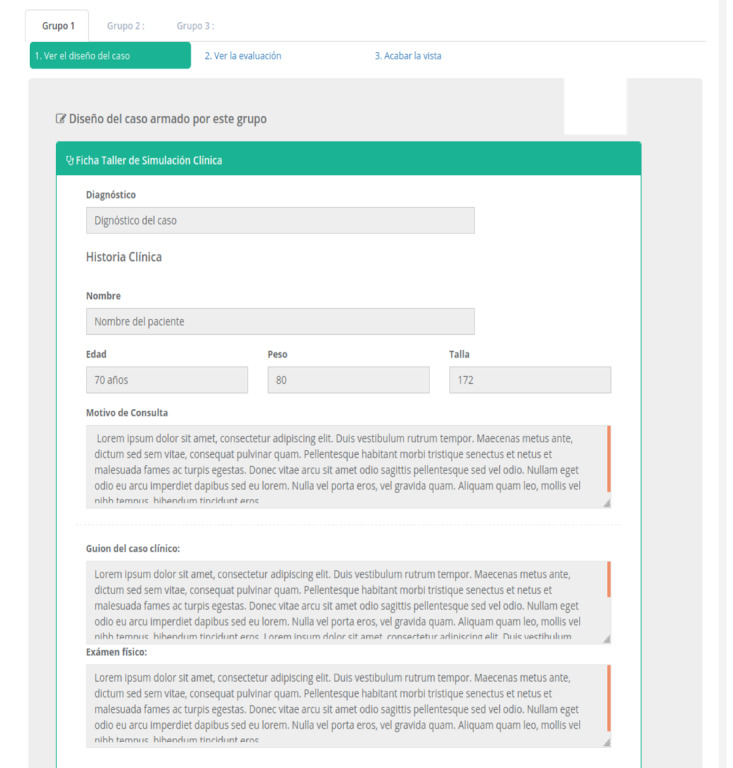
Teacher monitoring module. In this module, the teacher can monitor each group working in the collaborative design process in real time.

Throughout the students’ collaborative design process, the teacher supervises and facilitates the design process through the web platform via the internet, providing advice, clarifying doubts, and guiding each group in the preparation of the short clinical case scenario [15] (see Figure 7).

### Module 3: Collaborative Simulation

**Figure 8:**
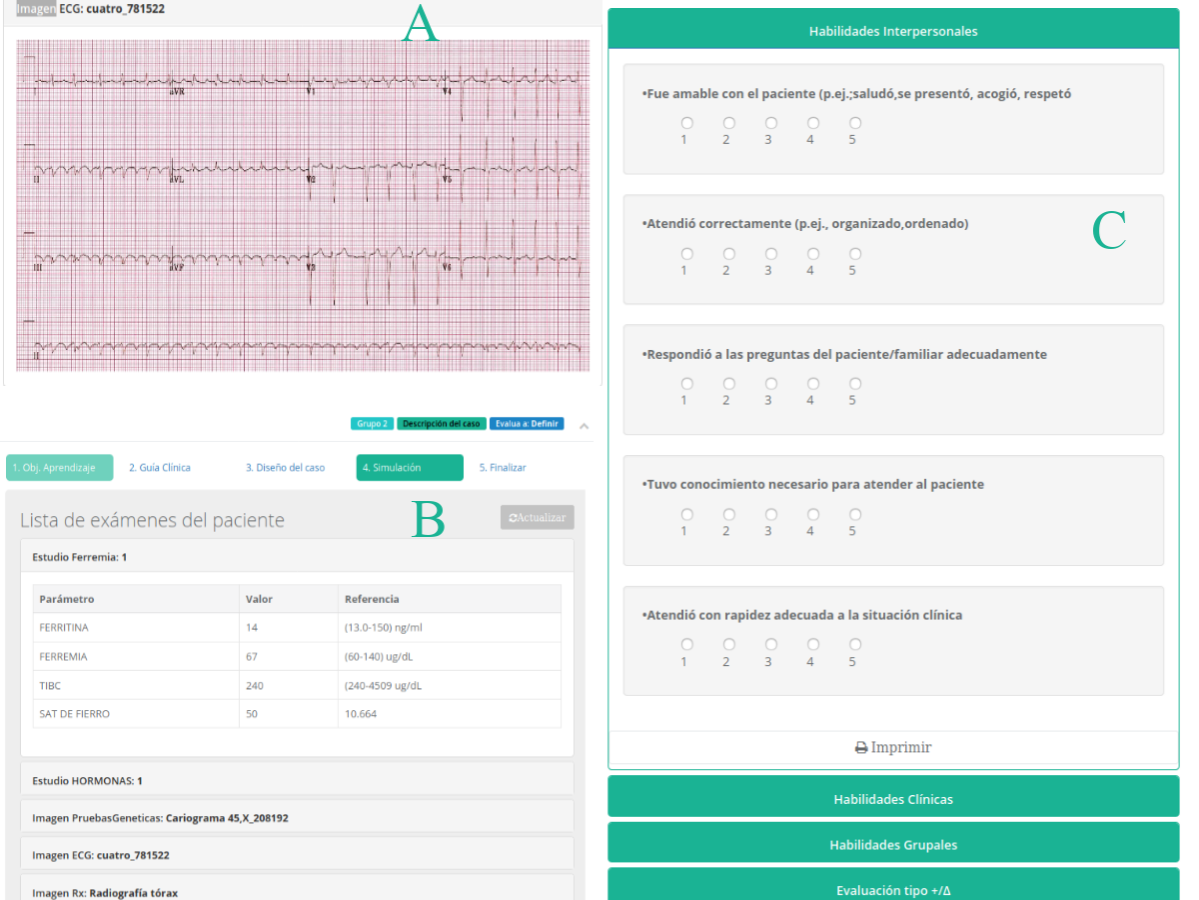
Collaborative simulation module. (A) and (B) are views of the performer group during the collaborative clinical simulation where an electrocardiogram and laboratory test are shown, respectively. (C) is a view of the assessment tool available for the evaluation of the performer group by the evaluator group. In the web platform, there are rubrics previously defined by the teacher that allow for assessment of the performance of the performer group in the simulation.

Collaborative simulation is a face-to-face activity that is executed in a simulated clinical environment, which provides a controlled and safe environment for the acquisition of clinical skills [34-36]. The members of the designer group prepare the scenario and simulate its case, perform roles, and present the case (ie, brief) to members of the executing group, who assume the role of the medical team. Members of the executing group, based on their knowledge and information, must obtain the anamnesis, perform a physical examination, request and interpret complementary exams and laboratory tests, diagnose, and treat the simulated patient [15] (see Figure 8).

All information is provided by the designer group as requested by the performer group when handling the patient inside the simulated room. The performer group may request laboratory results and exams, in images or video clips, and may visualize them on a desktop computer or mobile device. In parallel, the teacher controls the time and observes actions from a mirror room, ideally, and guides the designer group, which receives instructions through a hidden earpiece to help the performer group.

The third group, the evaluator group, observes the development of the case and evaluates the performance of the performer group in a separate room using the evaluation guidelines and the structured Plus/Delta assessment strategy in MOSAICO. This enables participants to consider the “pluses” (ie, what went well) and the “deltas” (ie, what they would like to change about the performance) in the web platform [37].

### Module 4: Collaborative Debriefing

**Figure 9:**
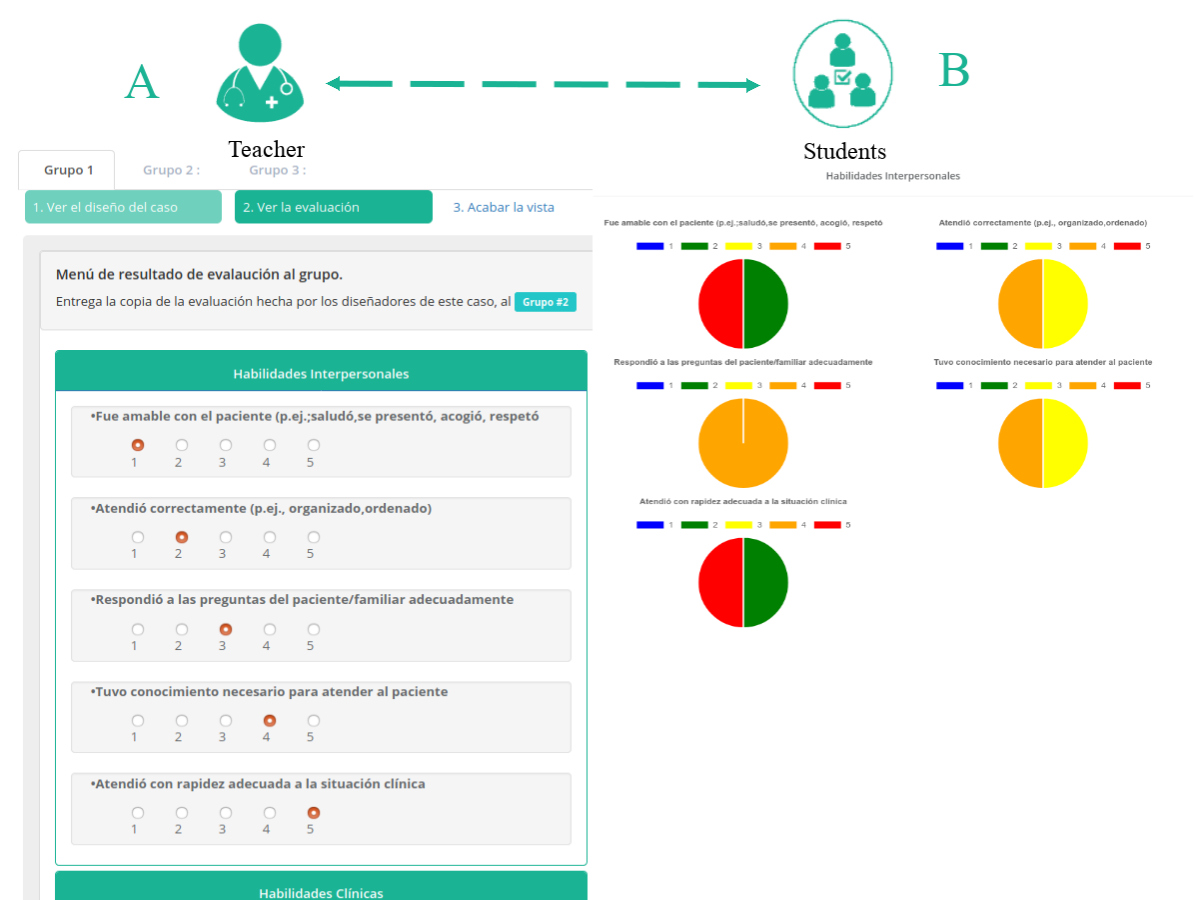
Collaborative debriefing module. Both (A) and (B) are views for the teacher. During the debriefing, the teacher can access the assessment done by each group. (A) shows the evaluations done for members of a group, while (B) shows summary charts of all the evaluations done for the students.

For the last phase of the CCS session, MOSAICO offers a summary module with all the logged activities, assessments by peers and the teacher, and video recordings for the three simulated clinical cases. This module is specially adapted so that the teacher and students can perform a collaborative debriefing, where each case is discussed deeply by the design, performance, and scoring carried out in the three groups (see Figure 9). At the end of the simulation, students can give, through the platform, their perception of the CSs and what they learned.

## Discussion

### Principal Findings

In this paper, a web platform, MOSAICO, is described in detail to facilitate the design, performance, and evaluation of collaborative clinical scenarios for medical students. MOSAICO was created to digitalize the collaborative simulation learning model that has been successfully applied for 4 years in the universities where the authors are based [15,17]. The web platform considers the CCS model’s functional and nonfunctional requirements and medical experts’ advice. The engineering software process (ie, design and development of the web platform) was oriented to support each phase of the model and extend its capabilities for designing clinical cases online via the internet.

The process of building a clinical case is a complex cognitive task. The students must work collaboratively to design a scenario representing a hypothetical clinical situation with enough fidelity to allow other students in a simulation phase to act collaboratively as a doctor team (ie, performer groups) to deduce a diagnosis and proceed to the medical actions. The designer group must coordinate to provide details like personal data, clinical history, laboratory or multimedia results, and vital signs to the performer group. According to simulation methodology, the last part of the session is used to analyze and discuss among all participants the experience and consequent relevant actions for solving the simulated situation in the real clinical practice. The teacher leads and stimulates reflection, allowing the students themselves to discover and assess their future behavior.

In the collaborative designer module, MOSAICO enables tools for team collaboration, supporting positive interdependencies among the team’s members as well as the diversity and depth of their clinical knowledge [38]. This is critical for the professional future of the medical students, where a physician does not work alone or is not isolated from a team. Instead, being part of a team has become a requirement, and leadership is a skill rather than a role [16].

Moreover, it is essential to include technologies that will be used in real-world settings into educational CSs to better prepare students for clinical practice and to promote patient safety [32]. In a recent perception study [39], European medical students believed that their curriculum lacked digital health education, with 84.9% (383/451) agreeing or strongly agreeing that it should be implemented in the medical curriculum. In Chile, the Universidad de Talca has implemented the CCS model since 2016 in the medical curriculum, creating the Biomedical Informatics course for medical students and funding the National Center for Health Information Systems (Centro Nacional en Sistemas de Información en Salud [CENS]) with four Chilean universities [40].

Recently at the CENS, we published a model of referential competencies in health information systems [41], which focuses on the health transformation and technology areas. These areas orient the design of curricula, training programs, and new careers associated with health and data science.

In this sense, MOSAICO is oriented to introducing electronic recording technologies and developing technological competencies in the curriculum of a medical faculty. Future employers expect new graduates to be competent in information technology use upon graduation. Students are often not given the significant educational opportunities needed to gain these competencies [32].

Assessing collaboration in undergraduate education is complex and requires special dedication [15]. How teamwork is measured and assessed is often a concern, making it difficult to include these skills in the undergraduate curricula [42]. The development of assessment instruments with reliability and validity based on statistical analyses should be designed and supervised by psychometricians, who propose items and dimensions to assess [15]. MOSAICO supports the construction and application of assessment instruments for CCS scenarios. In the educational design module, the teacher can design both their assessment guidelines and the one that students will apply to each other. The students apply this instrument in the collaborative simulation phase measuring the technical and nontechnical skills, registering all the items in the web platform for discussion in the debriefing phase. Understanding and developing teamwork is essential, and health care lags significantly behind fields such as the military and aviation [42]. In Chilean and Spanish medical faculties, as it happens in many other faculties, the students do not have an electronic registry that records the number of hours of CS that they had during their undergraduate studies.

For this reason, MOSAICO has the capabilities to maintain individual and group records of the simulated competencies, the assessments, and the detailed hours that each of the students had in the simulation center. This replicates the current aviation simulation models [42]. Besides, with the registration of cases and evaluations, investigations could be designed between different simulation centers, for both undergraduate and graduate medical students, which could allow for a better and fairer design of the end-of-program grade assessments of students’ performance.

The collaborative debriefing module is vital for enhancing learning and participating successfully in group discussions, with all the elements registered in the web platform. In this MOSAICO module, both teachers and students give feedback to learners and assess their participation in the activities [15]. Only 41% of the software programs used for medical training allow feedback to be given to students from the teacher or through automatic responses [13], but students work alone, before or after classes, without guidance along the learning process. The debriefing sessions are essential because they facilitate reflection, learning, conceptualization, abstraction, and connecting with real events [36].

The COVID-19 pandemic has significantly affected teaching within health career programs [10,43]; students have been without the possibility of doing rotations in hospitals, face-to-face classes, and CSs. In this scenario, MOSAICO has become a unique protagonist as a support web platform for the construction of online clinical cases, simulations via video conference, and debriefing with all the elements available (ie, cases, evaluations, and comments). Each stage in MOSAICO may happen in a different place, so the technology used should be flexible enough to support access from different devices, such as desktops or tablets, through the internet.

MOSAICO is growing; the next version (ie, version 2.0) should include more tools for the administration of simulation centers, modules for academic reports and supplies, different specialties (eg, psychiatry, obstetrics, and pediatrics), and improvement of the interoperability for the export of cases between different platforms or institutions.

During this online academic semester, MOSAICO is being evaluated by both teachers and medical students of the Universidad de Talca. For the evaluation, we are applying the mobile health (mHealth) App Usability Questionnaire (MAUQ) [44] to evaluate the usability of the web platform in mobile devices. This evaluation is a work in progress that considers two courses: (1) Semiology for third-year students (70 students in total) and (2) Medical Genetics for fifth-year students (30 students in total). In future work, we are planning to validate the application of MOSAICO in medical education, comparing it with similar platforms that use collaborative learning in CS. The application of MOSAICO in the CCS activities of cardiology courses at the Universität de Barcelona (120 students) and the Universidad de Vic-Manresa (64 students) in Spain will be assessed in a face-to-face or online modality, depending on the COVID-19 pandemic, during the next academic year.

### Limitations

The platform, which works on computers or mobile devices, is only available in Spanish. The next version will support both English and Spanish languages. MOSAICO supports a collaborative clinical session with three small groups composed of 3 to 5 students each. It does not allow the incorporation of another group in the same session, making it necessary to create another session if four or more groups are needed.

### Conclusions

MOSAICO was implemented online and is still currently being used correctly with different simulation sessions at the Universidad de Talca, Chile, where medical students work collaboratively connected by the internet. Both students and teachers have excellent comments about the use of the web platform. An essential strength of the platform is that it is possible to use it in face-to-face sessions or online via the internet without modifications.

The web platform supports all the stages of the CCS model satisfactorily, and the teachers use MOSAICO as technological infrastructure to schedule, design, and execute the simulation activities. Moreover, it allows for the teaching of clinical activities throughout the COVID-19 pandemic while the university campus is closed for student safety.

The use of the web platform simplifies the application and reuse of CS scenarios, permitting its use in multiple simulation centers. Moreover, its applications in different courses (ie, a large part of the medical curriculum) support the automatic tracking of simulation activities and their assessment.

MOSAICO could allow research to be conducted between different simulation centers by standardizing the information, structure of clinical cases, and assessment instruments. This is important in comparative studies and in research regarding medical students’ learning.

## Abbreviations

CCS: collaborative clinical simulation
CENS: Centro Nacional en Sistemas de Información en Salud (National Center for Health Information Systems)
CORFO: Corporación de Fomento de la Producción de Chile
CS: clinical simulation
CSCL: computer-supported collaborative learning
CSS 3: Cascading Style Sheets 3
GINMAD: Grup d’Innovació en Metodologies docents actives per el desenvolupament i avaluació de les competències clíniques en Medicina
MAUQ: mHealth App Usability Questionnaire
mHealth: mobile health
PHP: Hypertext Preprocessor
SQL: Structured Query Language

## References

[1] Edmunds S, Brown G (2010). Effective small group learning: AMEE Guide No. 48. Med Teach.

[2] Parmelee D, Michaelsen LK, Cook S, Hudes PD (2012). Team-based learning: A practical guide: AMEE guide no. 65. Med Teach.

[3] Tolsgaard MG (2013). Clinical skills training in undergraduate medical education using a student-centered approach. Dan Med J.

[4] Khan K, Pattison T, Sherwood M (2011). Simulation in medical education. Med Teach.

[5] Koschmann T, Koschmann T (1996). Paradigm shifts and instructional technology: An introduction. CSCL: Theory and Practice of an Emerging Paradigm.

[6] Tolsgaard MG, Kulasegaram KM, Ringsted CV (2016). Collaborative learning of clinical skills in health professions education: The why, how, when and for whom. Med Educ.

[7] Koops W, Van der Vleuten C, De Leng B, Oei SG, Snoeckx L (2011). Computer-supported collaborative learning in the medical workplace: Students' experiences on formative peer feedback of a critical appraisal of a topic paper. Med Teach.

[8] Lerner S, Magrane D, Friedman E (2009). Teaching teamwork in medical education. Mt Sinai J Med.

[9] Lethinen E, Hakkarainen K, Lipponen L, Rahikainen M, Muukkonen H (1999). Computer-Supported Collaborative Learning: A Review of Research and Development (The JHGI Giesbers Reports on Education, 10).

[10] Rose S (2020). Medical student education in the time of COVID-19. JAMA.

[11] Sandhu P, de Wolf M (2020). The impact of COVID-19 on the undergraduate medical curriculum. Med Educ Online.

[12] Ali M, Han SC, Bilal HSM, Lee S, Kang MJY, Kang BH, Razzaq MA, Amin MB (2018). iCBLS: An interactive case-based learning system for medical education. Int J Med Inform.

[13] Shahbazi B, Edalat-nejad M, Edalat-nejad N, Edalatnejad M (2012). Introduction of clinical, simulation-based software for medical sciences teachings. Procedia Eng.

[14] Schwarz D, Štourač P, Komenda M, Harazim H, Kosinová M, Gregor J, Hůlek R, Smékalová O, Křikava I, Štoudek R, Dušek L (2013). Interactive algorithms for teaching and learning acute medicine in the network of medical faculties MEFANET. J Med Internet Res.

[15] Guinez-Molinos S, Martínez-Molina A, Gomar-Sancho C, Arias González VB, Szyld D, García Garrido E, Maragaño Lizama P (2017). A collaborative clinical simulation model for the development of competencies by medical students. Med Teach.

[16] Paice E, Heard S (2003). Collaborative learning. Med Educ.

[17] Guinez-Molinos S, Maragaño Lizama P, Gomar-Sancho C (2018). Collaborative clinical simulation to train medical students [Article in Spanish]. Rev Med Chil.

[18] Dillenbourg P, Järvelä S, Fischer F, Balacheff N, Ludvigsen S, de Jong T, Lazonder A, Barnes S (2009). The evolution of research on computer-supported collaborative learning. Technology-Enhanced Learning.

[19] Tierney MJ, Pageler NM, Kahana M, Pantaleoni JL, Longhurst CA (2013). Medical education in the electronic medical record (EMR) era: Benefits, challenges, and future directions. Acad Med.

[20] Hallinger P, Wang R (2020). Analyzing the intellectual structure of research on simulation-based learning in management education, 1960–2019: A bibliometric review. Int J Manag Educ.

[21] Salas E, Bowers C, Rhodenizer L (1998). It is not how much you have but how you use it: Toward a rational use of simulation to support aviation training. Int J Aviat Psychol.

[22] Wiegers KE (2006). More About Software Requirements: Thorny Issues and Practical Advice.

[23] Freund J, Rücker B, Hitpass B (2017). BPMN Manual de Referencia y Guia Practica 5 Edicion: Con una Introducción a CMMN y DMN (Spanish Edition).

[24] Modeler. Camunda.

[25] Aurum A, Wohlin C (2005). Engineering and Managing Software Requirements.

[26] Wilbanks BA, Watts PI, Epps CA (2018). Electronic health records in simulation education: Literature review and synthesis. Simul Healthc.

[27] Sandars J, Lafferty N (2010). Twelve tips on usability testing to develop effective e-learning in medical education. Med Teach.

[28] Zahabi M, Kaber DB, Swangnetr M (2015). Usability and safety in electronic medical records interface design: A review of recent literature and guideline formulation. Hum Factors.

[29] Leff A, Rayfield J (2001). Web-application development using the model/view/controller design pattern. Proceedings of the Fifth IEEE International Enterprise Distributed Object Computing Conference.

[30] Maestre JM, Sancho R, Rábago JL, Martínez A, Rojo E, Moral ID (2013). Design and development of clinical simulation scenarios: Analysis of courses for anesthesiologists [Article in Spanish]. FEM (Ed. impresa).

[31] Hoerbst A, Ammenwerth E (2010). Electronic health records. A systematic review on quality requirements. Methods Inf Med.

[32] Kushniruk A, Borycki E, Kuo M, Parapini E, Wang SL, Ho K (2014). Requirements for prototyping an educational electronic health record: Experiences and future directions. Stud Health Technol Inform.

[33] Moreland R, Levine J, Wingert M, Witte EH, Davis JH (1996). Creating the ideal group: Composition effects at work. Understanding Group Behavior, Vol. 2. Small Group Processes and Interpersonal Relations.

[34] Dottin RL (2018). The Effects of Simulation-Based Training on Critical Thinking [doctoral dissertation].

[35] Ziv A, Small SD, Root Wolpe P (2000). Patient safety and simulation-based medical education. Med Teach.

[36] So HY, Chen PP, Wong GKC, Chan TTN (2019). Simulation in medical education. J R Coll Physicians Edinb.

[37] Motola I, Devine LA, Chung HS, Sullivan JE, Issenberg SB (2013). Simulation in healthcare education: A best evidence practical guide. AMEE Guide No. 82. Med Teach.

[38] Onal Vural M, Dahlander L, George G (2013). Collaborative benefits and coordination costs: Learning and capability development in science. Strateg Entrep J.

[39] Machleid F, Kaczmarczyk R, Johann D, Balčiūnas J, Atienza-Carbonell B, von Maltzahn F, Mosch L (2020). Perceptions of digital health education among European medical students: Mixed methods survey. J Med Internet Res.

[40] (2020). Centro Nacional en Sistemas de Información en Salud (CENS; National Center for Health Information Systems).

[41] Valderrama C (2019). A model of referential competences in health information systems: A Latin American perspective towards the definition of professional profiles. Proceedings of the AMIA 2019 Informatics Educators Forum.

[42] Britton E, Simper N, Leger A, Stephenson J (2015). Assessing teamwork in undergraduate education: A measurement tool to evaluate individual teamwork skills. Assess Eval High Educ.

[43] Sandhu P, de Wolf M (2020). The impact of COVID-19 on the undergraduate medical curriculum. Med Educ Online.

[44] Zhou L, Bao J, Setiawan IMA, Saptono A, Parmanto B (2019). The mHealth App Usability Questionnaire (MAUQ): Development and validation study. JMIR Mhealth Uhealth.

